# Synthetic Sex Pheromone in a Long-Lasting Lure Attracts the Visceral Leishmaniasis Vector, *Lutzomyia longipalpis*, for up to 12 Weeks in Brazil

**DOI:** 10.1371/journal.pntd.0002723

**Published:** 2014-03-20

**Authors:** Daniel P. Bray, Vicky Carter, Graziella B. Alves, Reginaldo P. Brazil, Krishna K. Bandi, James G. C. Hamilton

**Affiliations:** 1 Centre for Applied Entomology and Parasitology, Institute for Science and Technology in Medicine, Keele University, Keele, Staffordshire, United Kingdom; 2 Fundaçao Oswaldo Cruz, Instituto Oswaldo Cruz, Lab Doenças Parasitarias, Rio de Janeiro, Rio de Janeiro, Brazil; Fundaçao Oswaldo Cruz, Brazil

## Abstract

Current control methodologies have not prevented the spread of visceral leishmaniasis (VL) across Brazil. Here, we describe the development of a new tool for controlling the sand fly vector of the disease: a long-lasting lure, which releases a synthetic male sex pheromone, attractive to both sexes of *Lutzomyia longipalpis*. This device could be used to improve the effectiveness of residual insecticide spraying as a means of sand fly control, attracting *L. longipalpis* to insecticide-treated animal houses, where they could be killed in potentially large numbers over a number of weeks. Different lure designs releasing the synthetic pheromone (±)-9-methylgermacrene-B (CAS 183158-38-5) were field-tested in Araçatuba, São Paulo (SP). Experiments compared numbers of sand flies caught overnight in experimental chicken sheds with pheromone lures, to numbers caught in control sheds without pheromone. Prototype lures, designed to last one night, were first used to confirm the attractiveness of the pheromone in SP, and shown to attract significantly more flies to test sheds than controls. Longer-lasting lures were tested when new, and at fortnightly intervals. Lures loaded with 1 mg of pheromone did not attract sand flies for more than two weeks. However, lures loaded with 10 mg of pheromone, with a releasing surface of 15 cm^2^ or 7.5 cm^2^, attracted female *L. longipalpis* for up to ten weeks, and males for up to twelve weeks. Approximately five times more sand flies were caught with 7.5 cm^2^ 10 mg lures when first used than occurred naturally in non-experimental chicken resting sites. These results demonstrate that these lures are suitably long-lasting and attractive for use in sand fly control programmes in SP. To our knowledge, this is the first sex pheromone-based technology targeting an insect vector of a neglected human disease. Further studies should explore the general applicability of this approach for combating other insect-borne diseases.

## Author Summary

Visceral leishmaniasis is a neglected disease of increasing worldwide importance. In Brazil, the protozoan parasites which cause visceral leishmaniasis are transmitted through blood-feeding of the female sand fly *Lutzomyia longipalpis*. As no human vaccine is available against VL, new tools for targeting sand flies are urgently needed to reduce transmission. Here, we develop and test a new tool for controlling *L. longipalpis*: a long-lasting lure, which releases a synthetic sex pheromone that attracts both male and female *L. longipalpis*. Through a series of field experiments conducted in São Paulo state, we show that these lures can attract female *L. longipalpis* for up to 10 weeks from first use, and males for up to 12 weeks. This technology could be applied by disease control agencies to attract sand flies away from feeding on people and their animals, and towards insecticide treated surfaces where they can be killed. To our knowledge, this is the first example of a feasible insect sex pheromone technology that could be applied as part of a programme designed to protect against a human disease.

## Introduction

Visceral leishmaniasis (VL) remains one of the world's most important neglected diseases, with over 350 million people estimated to be at risk of infection [1]. Despite on-going work, there is no human vaccine to protect against the causative agents, protozoan parasites of the genus *Leishmania*, and the disease is usually fatal if left untreated [2], [3]. Most efforts to control VL in Brazil, where the disease is endemic in many parts of the country, have focused on culling infected dogs, the primary mammalian reservoir of the causative agent *Leishmania infantum* [4], [5]. This practice, which is difficult to implement effectively, has not prevented the spread of the disease from rural into urban or urban to urban areas [6], [7]. New tools, less reliant on dog culling, are therefore urgently needed to reduce the number of cases of VL, and prevent further spread of the disease.

In some cases, protection against leishmaniasis can be achieved by targeting the blood-feeding sand fly vectors of the disease using insecticides [8]. In Brazil, *L. infantum* is transmitted between vertebrate hosts by blood-feeding female *Lutzomyia longipalpis*. Male *L. longipalpis* do not blood-feed, but produce sex pheromones which, in combination with host odour, attract females to mating aggregations formed on or above animals at dusk [9], [10]. Chicken sheds are common aggregation sites, offering *L. longipalpis* both a supply of animals on which to blood-feed, and suitable surfaces (walls and perches) on which to court and mate. Although high numbers of sand flies can occur in chicken sheds, chickens are refractory to leishmaniasis, and therefore do not contribute directly to transmission [11].

Spraying chicken sheds with residual insecticides may seem a logical strategy for *L. longipalpi*s control, and does reduce numbers of sand flies in animal houses [12]. However, by disrupting pheromone production by killing male *L. longipalpis*, it also leads to diversion of blood-seeking females away from treated chicken sheds, and potentially towards dogs and people [12], [13]. Spraying of chicken sheds might therefore accidentally increase VL transmission. In addition, because of the large number of potential aggregation sites in Brazil, it is impossible to treat them all with insecticide. In the state of São Paulo, for example, insecticide spraying is only performed in a 200 m radius following a human case [14].

Recently, we have shown that it is feasible to improve sand fly control through use of a synthetic version of the *L. longipalpis* sex pheromone (*S*)-9-methylgermacrene-B. The synthetic pheromone, (±)-9-methylgermacrene-B (CAS 183158-38-5), attracts both male and female *L. longipalpis*, and prevents diversion of both sexes away from insecticide-treated chicken sheds [13], [15]. Properly formulated, the synthetic sex pheromone could attract blood-feeding female *L. longipalpis* away from feeding on people and dogs and towards insecticide-treated sheds where they can be killed. This technology could therefore greatly increase the effectiveness of insecticide spraying in animal houses as a means of managing local sand fly populations and thereby reduce *Le. infantum* transmission.

Previous experiments demonstrating the efficacy of the synthetic pheromone were conducted using a simple device designed to release the attractant over a single night [13]. In order for this technology to be applied by disease control agencies however, a pheromone lure would have to remain effective over a period of weeks or months prior to replacement. Ideally, the pheromone would be released for as long as the insecticide treatment remained active, so that both could be replaced simultaneously. In addition, in order to attract both males and females to insecticide-treated sheds, the synthetic pheromone lure must compete successfully against the levels of pheromone released by males from untreated aggregations sites.

The aim of this study was to develop a synthetic sex pheromone lure that could attract *L. longipalpis* over a prolonged period of time in the field, and could therefore be used by health authorities as part of leishmaniasis control programmes. We attempted to increase the length of time over which our prototype lures were effective by increasing the amount of pheromone loaded into each device, and optimizing the surface area from which the pheromone was released. We then measured the length of time over which different lure types were effective in attracting sand flies to experimental chicken sheds in the field, with reference to numbers caught in both real (i.e. homeowner constructed sheds) and experimental sheds without pheromone lures. Furthermore, to demonstrate the effectiveness of this technology over a wider geographical area, experiments presented here were conducted in Araçatuba, São Paulo state, 450 km from our original testing site in Campo Grande (Mato Grosso do Sul). *L. longipalpis* and leishmaniasis are spreading toward São Paulo city, one of the largest cities on Earth, and considered to be the economic epicentre of Brazil [16]. It is therefore crucial that new methods of sand fly control are tested for use in this State.

## Methods

### Field site

Field studies were conducted in Araçatuba, a municipality of 180,000 inhabitants [17], located in Western SP state (21°12′32″S 50°25′58″W), approximately 470 km NW of São Paulo city. Araçatuba is surrounded by a region of smaller towns and villages, separated by farmland. The climate is classified as tropical with a dry winter [18]. *L. longipalpis* was first reported from Araçatuba in 1997 [19], and males of this population produce the pheromone (S)-9-methylgermacrene-B [18]. Following the report of the first human case from the area in 1999, visceral leishmaniasis is now considered to be endemic in Araçatuba [20].

All experiments were conducted in private gardens and yards, which typically consisted of a walled-in area, located at the front or back of a house, containing fruit trees and animal shelters. Gardens were chosen on the basis that they had chickens and that *L. longipalpis* were present and which could be confirmed through preliminary capture with miniature light traps [21].

### Sand fly survey

To ascertain numbers of *L. longipalpis* aggregating naturally near chickens in Araçatuba, we carried out a small-scale survey using miniature light traps. Trapping was conducted between January–May in 2012, which represents the middle of the sand fly season in Araçatuba, between the start of rains in October and the beginning of the dry winter in July. Light traps powered by a 12 V battery were placed in chicken sheds or other chicken resting sites (e.g. trees) for 1–2 nights per household. To facilitate comparisons with results of experiments using pheromone, numbers of sand flies caught were expressed as, average number of males and females caught per trap per night in each garden.

While *L. longpalpis* is the known vector of visceral leishmaniasis in Brazil, several other species of sand fly, including vectors of cutaneous leishmaniasis, also occur in the state of São Paulo [22]. To check for the presence of species other than *L. longipalpis* in our study area, a subset of sand flies caught in gardens and experimental chicken sheds were identified to species level through inspection of genitalia [23].

In an attempt to measure the number of sand flies feeding on dogs in Araçatuba, we used miniature light traps to sample in the vicinity of 12 pet dogs, which regularly slept outside, and were therefore at risk of sand fly biting. At dusk, dogs were secured inside a metal wire cage (60 cm (w) ×78 cm (h) ×92 cm (l)) containing bedding, food and water at the site where they normally slept. A miniature light trap was mounted above the cage inside an upturned cylindrical plastic container (62 cm (h) ×53 cm (d)) that covered most of the upper surface of the cage. The light in the trap was only visible from below, and not through the sides of the plastic container. This design allowed for sand flies feeding and mating on the dog to be attracted upwards towards the trap, without the light attracting additional flies to the dog cage. Dogs remained in cages until the following morning, when traps were collected, dogs released, and sand flies counted.

### Experiment 1. Testing of synthetic pheromone for sand fly attraction in Araçatuba

The goal of the first experiment was to determine whether the synthetic sand fly sex pheromone (±)-9-methylgermacrene-B attracted both male and female *L. longipalpis* in SP state. Numbers of sand flies caught in experimental chicken sheds with pheromone were compared to numbers caught in paired control sheds without pheromone. This design has previously demonstrated attraction to the pheromone in Campo Grande, MS [15].

Experimental chicken sheds were constructed from four panels of plywood (55 cm (w) by 105 cm (h) by 1.5 cm thick). These 4 panels were placed so as to form an open topped box, the sides of which were held together by plastic garden ties inserted through holes (15 mm ID) drilled into the four corners of each panel. In addition, each panel had three holes (1 cm ID) drilled in a horizontal line across the middle to facilitate release of host odour from the box. Two panels of each box placed opposite each other had a small notch cut out of the centre of the top edge of the panel into which a wooden pole was placed and from which a miniature CDC light trap was suspended.

Prior to sunset, two pairs of sheds were mounted 3 m apart in a large garden in Araçatuba, with a distance of 10 m between pairs. A chicken was placed into each shed, as a combination of host odour and pheromone is required to attract female sand flies [15]. In the test shed, ten pheromone lures, consisting of 2.5 cm×2.5 cm sealed 6.25 cm^2^ square plastic pouches each loaded with 50 µg of synthetic (±)-9-methylgermacrene-B, were attached to the underside of the light trap lid. Each lure releases an amount of pheromone equivalent to a group of 50 males [15]. Ten lures were used as this resulted in greater numbers of males caught compared to a single lure [13]. The control trap in each pair was similarly fitted with 10 control lures containing no pheromone.

The following morning, traps were removed, the chickens released, and sand flies caught in each shed placed into a −20°C freezer until dead. The numbers of male and female sand flies caught in test and control sheds were then counted under a microscope (Quimis Ltda., São Paulo) at ×20 magnification. A total of twelve replicates were performed over six nights. To control for any positional bias in numbers of sand flies caught, the positions of test and control sheds in each pair was reversed between replicates.

### Experiment 2. Longevity of lures containing 1 mg synthetic pheromone

The lures used in Experiment 1 were designed to release pheromone over one night only [15]. The goal of Experiment 2 was to develop and test the longevity of a new lure, made of thicker plastic than those used in Experiment 1, designed to last several weeks. Each lure consisted of a 6×2.5 cm sealed pouch with a surface area of 30 cm^2^, loaded with 1 mg of synthetic pheromone. In order to increase the longevity of the lures, the surface area from which pheromone could be released was reduced using electrical tape. Either 75% or 50% of the pouch was covered with the non-permeable tape, resulting in lures with releasing surfaces of 7.5 cm^2^ and 15 cm^2^ respectively. All lures were stored at −20°C, and removed from the freezer 24 h prior to use to allow pheromone release rate to equilibrate before testing [24].

Field-testing was conducted between December 2011 and August 2012 using a modification of the protocol used in Experiment 1. To take into account that lures were loaded with more pheromone, the distance between test and control sheds was increased to 5 m to reduce the chances of a lure in a test shed attracting sand flies to a nearby control shed. Similarly, to prevent interference between pairs, only a single pair of sheds was mounted in each garden. For each replicate, a single 1 mg lure (either 7.5 cm^2^ or 15 cm^2^ releasing surface) was attached to the trap in the test shed, with no lure attached to traps in control sheds. Both sheds contained a chicken as before. In the morning, chickens and lures were removed, and numbers of sand flies counted.

Lures were tested when new and at fortnightly intervals for up to 8 weeks from first use. Between replicates, lures were kept outside in a shaded area at our field station in Araçatuba. Experiments were typically conducted for a maximum of four nights in any given garden (using both lure types) before changing locations, changing control/test box positions each night.

### Experiment 3. Longevity of lures containing 10 mg synthetic pheromone

The goal of Experiment 3 was to determine whether the longevity of the new lure design could be further increased by loading more synthetic pheromone into each pouch. Repeating the protocol described in Experiment 2, we tested lures loaded with 10 mg of sex pheromone during the same field season. In order to better understand the effect of the size of the releasing surface on lure longevity, we tested 10 mg lures with three different surface areas. In addition to the 7.5 cm^2^ and 15 cm^2^ lures used in Experiment 2, we also tested lures that had not been covered with electrical tape, with releasing surfaces of 30 cm^2^. Lures were tested when new, and at fortnightly intervals up to twelve weeks after first use.

A final test was carried out to confirm that decreases in attraction observed with aged lures were not a consequence of changes in sand fly numbers at the end of the field season. Therefore, 12 new 10 mg lures with 7.5 cm^2^ releasing surfaces were tested the week following the final tests with the 12 week old lures in Experiment 3.

### Data analysis

Numbers of sand flies caught in light traps were best described as following an over-dispersed pattern of distribution. In order to reduce this dispersion, numbers of sand flies caught in test and control boxes were log+1 transformed (treating males and females separately) prior to analysis [25]. While this reduced the influence of outliers (traps catching large number of sand flies), and increased symmetry around sample medians, it did not in all cases result in distributions considered suitable for analysis using parametric tests. Therefore, Wilcoxon signed-ranked tests performed on log+1 transformed data were performed to determine whether lures in test traps caught significantly more sand flies than paired controls. This procedure was used to determine whether synthetic sex pheromone was attractive to *L. longipalpis* in Araçatuba (Experiment 1), and whether each type of long lasting lure was significantly attractive at each time point tested (Experiments 2 and 3). In all cases, numbers of sand flies caught in each test trap overnight were compared with numbers in the paired control on the same night. Numbers of male and female *L. longipalpis* caught were treated separately. Where one or both of the light traps failed overnight, numbers of sand flies caught in both the test and control trap that night were excluded from analysis. All analyses were performed in R, version 2.14.2 [26], with estimates of aggregation (k) [27] derived using the MASS package [28].

### Ethical statement

The use of chickens, dogs and private gardens for experiments was conducted with the informed consent of homeowners. The trial of which this work is part was reviewed by the Comissão de Ética no Uso de Animais (CEUA) and the Comitê de Ética em Pesquisa (CEP) at Fiocruz, Rio de Janeiro. We follow the guidelines which are established in Brazilian Law No. 11.794, of 08.10.2008. We also have ethical approval from the Ethical Review Panel at Keele University, UK and follow the ethical rules of the UK Home Office. These rules are governed by the Animals (Scientific Procedures) Act 1986. In addition, as we are funded by the Wellcome Trust, we comply with the Common Rules for Animal Research which are prepared by the UK National Centre for the Replacement, Refinement and Reduction of Animals in Research (NC3Rs). These rules require us, when collaborating with laboratories outside the UK, to check that welfare standards are consistent with the principles in UK legislation and guidelines.

## Results

### Sand fly survey

Throughout the study, 772 light trap captures were performed in 59 private gardens. This includes light trap captures for sand fly surveying, experiments 1–3, and captures above dogs. Sand flies were caught in miniature light traps placed in 30/59 (50.8%) of gardens surveyed. The distribution of both sexes was aggregated across sampled locations (i.e. relatively large numbers of sand flies caught in a small proportion of surveyed gardens: males: mean per garden = 3.45, k = 0.22; females: mean per garden = 0.72, k = 0.39). Where sand flies were caught in gardens, males were approximately three times more abundant than females (median males caught (interquartile range) 2.50 (1.00–7.80), females 0.83 (0.00–2.13).

A total of 5 male and 2 female sand flies were caught above 4 of 12 dogs sampled. No sand flies were caught above the remaining 8 dogs.

Male (n = 81) and female (n = 22) sand flies caught in light traps throughout the study period were identified to species level through inspection of genitalia. All were confirmed to be *L. longipalpis*.

### Experiment 1. Testing of synthetic pheromone for sand fly attraction in Araçatuba

**Figure 1:**
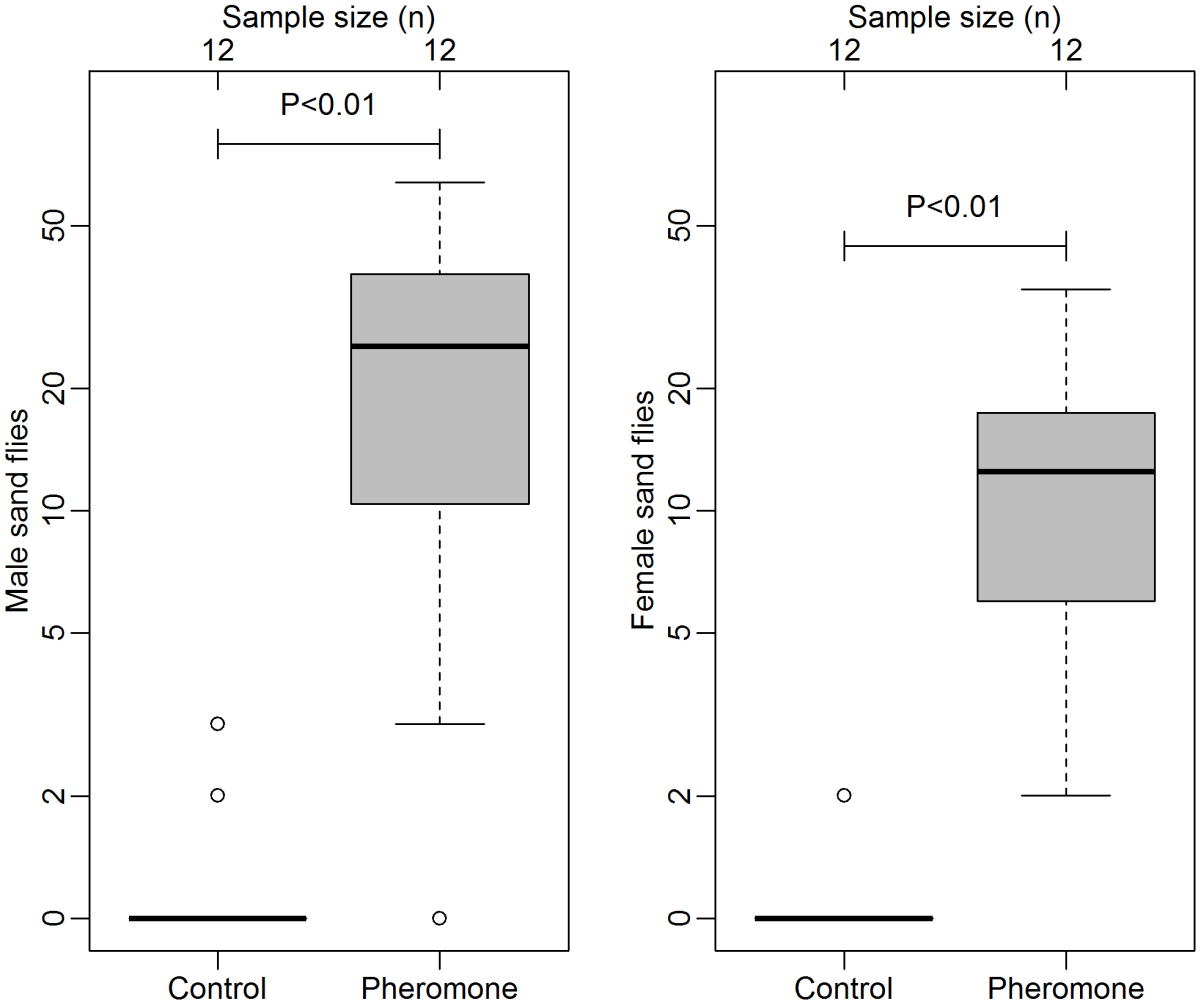
Number of male (left side) and female (right side) *Lutzomyia longipalpis* attracted per night to experimental chicken sheds with either 10 control lures (no pheromone) or 10 pheromone lures each loaded with 50 µg of synthetic sand fly pheromone. Box and whisker plots show median (horizontal line), interquartile range (box), maximum extreme value within 1.5 of interquartile range (whiskers) and outliers (open circles). Data log(+1) transformed prior to analysis (Wilcoxon signed rank test).

Significantly more male sand flies were attracted to experimental chicken sheds with 0.5 mg (10×50 µg) pheromone lures (median (interquartile range) = 24.50 (10.25–34.75)) than controls sheds without pheromone (0.00 (0.00–0.00), Wilcoxon signed rank test on log(+1) transformed data, P<0.01, Fig. 1). Similarly, more females were attracted to sheds with pheromone (11.50 (5.00–5.75)) than those without (0.00 (0.00–0.00), P<0.01, Fig. 1). These results demonstrate that synthetic pheromone, presented in combination with naturally-released host odour, is significantly attractive to both male and female *L. longipalpis* in Araçatuba. In comparison, light traps placed overnight in the two existing chicken sheds in the same garden prior to the beginning of the experiment caught a total of 9 male and 4 female *L. longipalpis*.

### Experiment 2. Longevity of lures containing 1 mg synthetic pheromone

**Figure 2:**
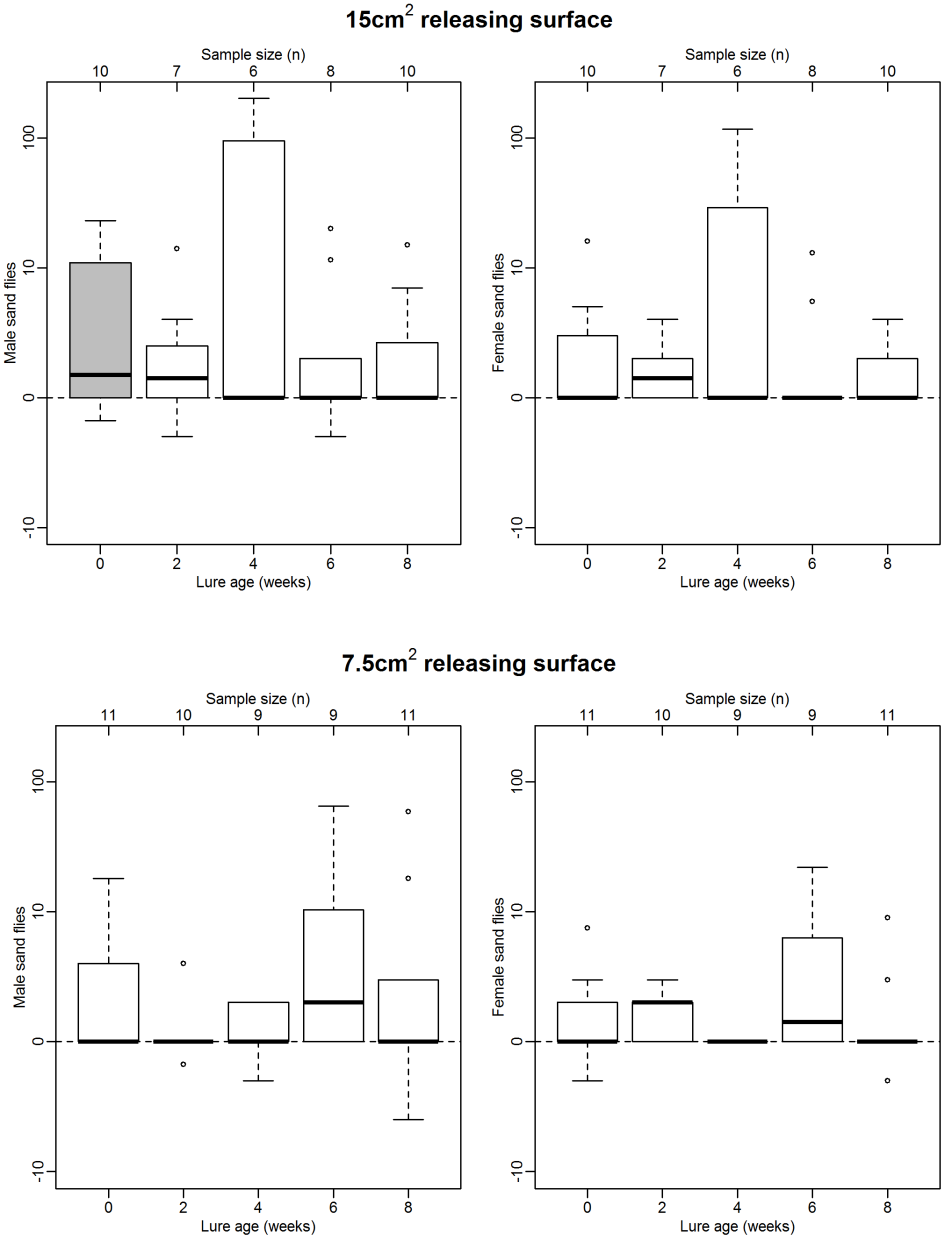
Attractiveness of lures loaded with 1*L. longipalpis* caught in test sheds with pheromone lures minus number caught in paired control sheds with no pheromone. Box and whisker plots show median (horizontal line), interquartile range (box), maximum extreme value within 1.5 of interquartile range (whiskers) and outliers (open circles). Grey boxes indicate significantly more sand flies caught in test sheds than controls (P<0.05, Wilcoxon signed rank test, data log+1 transformed prior to analysis).

Significantly more male sand flies were attracted to test sheds containing new 1 mg 15 cm^2^ lures than to control sheds without lures. (test: 2.00 (0.50–10.00), control: 0.00 (0.00–1.50), P<0.05, Fig. 2, top left). However, these same lures did not attract significantly more females than control sheds, even when new (test: 0.00 (0.00–2.00), control: 0.00 (0.00–0.00), NS, Fig. 2, top right). After two weeks in the field, numbers of both males and females attracted to test sheds were not significantly different to those at controls, indicating that the 1 mg 15 cm^2^ lures were no longer attractive to either sex.

Lures with 1 mg of pheromone and a smaller releasing surface of 7.5 cm^2^ were not attractive, when new or otherwise, to both males (test: 0.0 (0.00–3.75), control: 0.00 (0.00–0.00), NS, Fig. 2, bottom left), or females (test: 0.00 (0.00–3.75), control 0.00 (0.00–0.00), NS, Fig. 2, bottom right). These results suggest that not enough pheromone was released from lures loaded with 1 mg to attract either sex of *L. longipalpis* in the field.

### Experiment 3. Longevity of lures containing 10 mg synthetic pheromone

**Figure 3:**
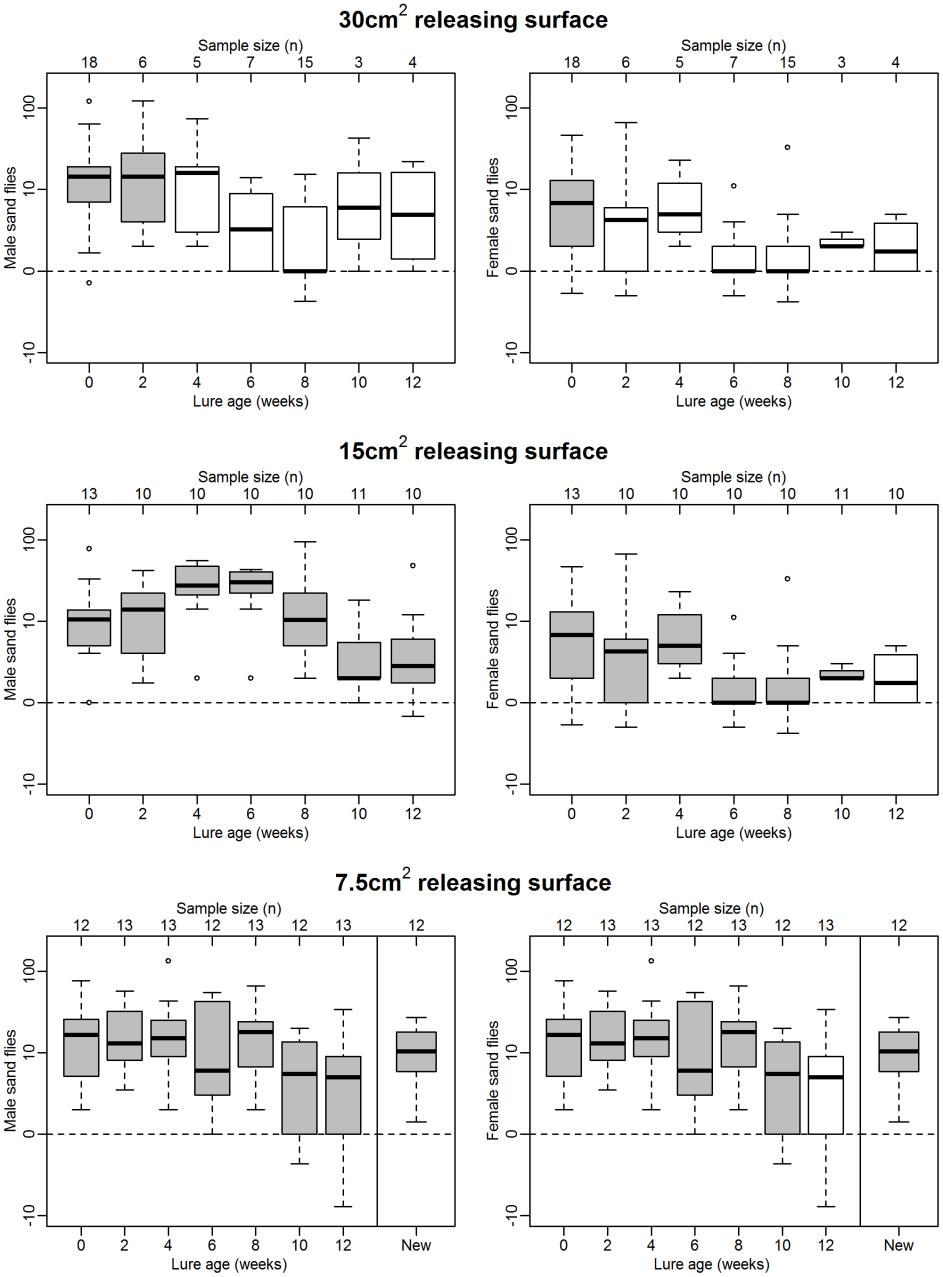
Attractiveness of lures loaded with 10*L. longipalpis* caught in test sheds with pheromone lures minus number caught in paired control sheds with no pheromone. Box and whisker plots show median (horizontal line), interquartile range (box), maximum extreme value within 1.5 of interquartile range (whiskers) and outliers (open circles). Grey boxes indicate significantly more sand flies caught in test sheds than controls (P<0.05, Wilcoxon signed rank test, data log+1 transformed prior to analysis). ‘New’ refers to fresh lures tested one week after 12 week old lures, to confirm that significant numbers of *L. longipalpis* could still be caught at the end of the sand fly season.

When first used in the field, lures loaded with 10 mg of synthetic pheromone with a surface area of 30 cm^2^ attracted significant numbers of both male (test: 25.0 (11.25–55.50), control: 0.25 (0.00–0.50), P<0.001, Fig. 3, top left) and female *L. longipalpis* (test: 9.00 (3.25–13.50), control: 0.00 (0.00–1.00), P<0.001, Fig. 3, top right). However, these lures only continued to attract male sand flies for up to 2 weeks in the field, and only new lures attracted significant numbers of females. Therefore, while an improvement in longevity compared to 1 mg lures was achieved by increasing the amount of pheromone loaded, it was relatively short-lived.

Reducing the releasing surface of 10 mg lures, however, resulted in further improvements. When first used in the field, 10 mg lures with a releasing surface of 15 cm^2^ attracted significant numbers of males (test: 18.00 (6.00– 32.00), control: 0.00 (0.00–1.00), P<0.001, Fig. 3, middle left) and females (test: 4.00 (1.00–6.00), control: 0.00 (0.00–0.00), P<0.01 Fig. 3, middle right). This attraction continued in males for up to 12 weeks, and in females for 10 weeks. Similarly, lures with a releasing surface of 7.5 cm^2^ also attracted significant numbers of males (test: 18.00 (12.75–27.75), control: 0.00 (0.00–0.00), P<0.01 Fig. 3, bottom left) and females (test: 4.00 (0.75–6.00), control: 0 (0.00–0.00), P<0.01, Fig. 3, bottom right) when first used. These lures also continued to attract males for up to 12 weeks, and females for ten weeks.

New 7.5 cm^2^ lures tested at the end of the experimental period caught significant numbers of male (test:18.50 (14.75–28.00), control: 1.00 (0.00–1.25), P<0.01, Fig. 3, bottom left) and female *L. longipalpis* (test: 4.00 (1.00–5.50), control: 0.00 (0.00–0.00), P<0.01, Fig. 3, bottom right). This last result indicates that decreases in the number of sand flies in Experiment 3 occurred as a result of lure age, rather than a lack of sand flies at the end of the season.

## Discussion

The use of synthetic sex pheromones, as a means of attracting insects is now commonplace in agriculture, and is a key component of many integrated management systems targeting crop pests [29]. However, despite this success, little progress has been made in developing similar sex pheromone-based tools effective against biting vectors of animal and human diseases. While host odours have been trialled as a means of attracting or repelling biting insects (e.g. midges [30], [31]), the use of sex pheromones as potential tools for control has been almost entirely overlooked. This may in part be due to the difficulty associated with isolating and identifying sex pheromones, which are often produced in very small amounts by insects. In addition, sex pheromones may comprise one or more components, some of which may be new to science (as was the case for *L. longipalpis* [32]). However, the advantage of using sex pheromones is that they are specific to an individual species, attracting the target insect only, with minimal effect on other invertebrate fauna.

Here, we have demonstrated that a lure releasing the synthetic sex pheromone (±)-9-methylgermacrene-B attracts the leishmaniasis vector *L. longipalpis* to chicken sheds for up to 10 to 12 weeks. To our knowledge, this is the first time a sex pheromone-based technology, feasibly long-lasting for use as a control measure, has been developed and shown to be effective against an insect vector of human disease. This lure could be deployed in a number of ways to improve the efficiency of current strategies targeting *L. longipalpis*. In agriculture, pheromone lures attached to sticky traps are used as a sensitive means of monitoring for the presence of target species [29]. This information is used by farmers to decide when to treat crops with insecticide, prior to further growth of the pest population. Similarly, sticky traps fitted with synthetic pheromone lures could provide a lower cost alternative to battery powered miniature light traps for sand fly monitoring. Previous studies have demonstrated that the synthetic pheromone can attract both sexes of *L. longipalpis* to sticky traps, and that host odour is not required to catch males [13], [15]. Widespread use of such a trap could aid disease control agencies in identifying areas where *L. longipalpis* is present, and allow intervention strategies to target high risk areas prior to the onset of human VL cases.

Beyond use as a tool for monitoring, the greatest impact that the pheromone could have on *L. longipalpis* population management is in improving the effectiveness of insecticide spraying. *L. longipalpis* remain broadly susceptible to residual insecticides [33], but potential diversion of sand flies away from sprayed sheds, coupled with the cost of repeated insecticide application, severely limits the cost-effectiveness of this strategy. Although biting females which transmit *Leishmania* through blood-feeding would be considered the primary target for a pheromone-enhanced insecticide intervention, attracting males to killing sites would prevent both sexes being diverted to non-treated aggregation sites elsewhere [12].

The sex pheromone lure could most effectively be deployed in conjunction with a long-lasting killing agent, such as a micro-encapsulated formulation of a residual insecticide, or insecticide-impregnated netting [34]. Previous tests have shown that the sex pheromone can attract both sexes to experimental sheds treated with micro-encapsulated lambda-cyhalothrin [13]. The efficacy of this formulation against sand flies diminished by only 12% over 3 months when applied to outdoor surfaces [35]. It is therefore feasible that the insecticide and lure could be renewed/refreshed by a public health technician, for example once every three months, in a single visit. Further studies are needed to determine the optimal timing of such an intervention: perhaps at the beginning of the rainy season, when sand fly numbers are low. In addition, some consideration may need to be given as to whether targeting *L. longipalpis* over prolonged periods could lead to growth in populations of other sand fly disease vectors, through reduced competition. Other species of sand fly have been recorded from the Araçatuba region [22], although none were recovered here in our small-scale survey. A suitable protocol would also have to minimize the risk of *L. longipalpis* developing insecticide resistance as a consequence of increased exposure through attraction to insecticide treated sheds.

Lures with a surface area of 7.5 cm^2^, loaded with 10 mg of pheromone, attracted a median of 18 more males and 9 more females per night when new to test sheds, compared to control sheds without pheromone. Throughout the study, control sheds typically attracted zero sand flies. While this difference may seem modest, these figures represent approximately five times the number of sand flies occurring naturally near chickens in trees and sheds in Araçatuba (as determined by our light trap survey), and many more than we were able to catch near dogs. Comparisons of sand fly numbers may therefore indicate that a shed treated with a pheromone lure could compete with attraction to existing aggregation sites. This technology may therefore have the potential to lure sand flies away from current aggregations, towards sheds treated with insecticide. If so, it would not be necessary to treat all aggregation sites in an area, increasing the cost effectiveness and sustainability of insecticide spraying as a strategy for *L. longipalpis* control. Despite the relatively low numbers of sand flies caught in Araçatuba, more than 30% of dogs in some areas of the city are seropositive for *Leishmania* [36]. This may indicate a relatively high proportion of *L. longipalpis* within the city are infected with the parasite.

Although the male-produced *L. longipalpis* pheromone attracts both sexes, we consider it to be a sex pheromone because conspecific males are only attracted for the purpose of mating [37]. The ratio of males to females caught (approximately 4∶1) was similar in both experimental sheds with pheromone and natural sand fly aggregation sites in trees and chicken sheds. This difference between sexes was also observed in previous studies in Campo Grande using both light traps and non-powered sticky traps [15]. This effect may occur as a consequence of sex-specific differences in behaviour [10]: male *L. longipalpis* arrive earlier at aggregation sites than females and stay longer, increasing their chances of capture. Lures also remained attractive to males for a longer period than females, perhaps indicating that the female attractive response is more sensitive to the amount of pheromone released. In addition, there may be fewer females available to be captured from the environment than males. It would therefore be more difficult to detect a significant difference between numbers of females caught at test and control stations, compared to males, with the same number of experimental replicates.

Comparisons of different lure designs demonstrated that lure longevity could be increased through loading of larger amounts of sex pheromone into the device, and reducing the surface area from which the attractive chemical was released. However, no additional increase in longevity was achieved by decreasing the surface area beyond 15 cm^2^. Further longevity might be achievable by increasing the amount of pheromone in each lure beyond 10 mg, although a trade-off will exist between the financial costs of loading each lure with more pheromone versus the benefits in terms of additional lifespan. While lures designed to last a single night loaded with 50 µg of pheromone were attractive to both sexes of *L. longipalpis*, the ‘longer lasting’ design loaded with 1 mg of pheromone were not effective, attracting only males when first used. This may indicate that, despite the higher pheromone load, the pheromone release rate from the long lasting design was not sufficient to attract female sand flies.

*L. longipalpis* is a species complex: males from different reproductively isolated populations producing different pheromones, with (S)-9-methylgermacrene-B the most geographically widespread [38]. We have shown that synthetic (±)-9-methylgermacrene-B is attractive to *L. longipalpis* in both Mato Grosso do Sul [15] and São Paulo state, and is therefore likely to be effective across those parts of South America and Central where this chemotype occurs. Nevertheless, efforts should be made to synthesize pheromones of different sand fly populations. These include 3-methyl-α-himachalene, released by *L. longipalpis* in North East Brazil, where large numbers of human cases of visceral leishmaniasis occur [38]. There are also sand fly vectors of cutaneous leishmaniasis that are suspected of producing sex pheromones, which could be targeted through a similar approach [39]. Furthermore, identifying the components of host odour which synergize attraction to pheromone [40] should be a priority. This would facilitate the development of a standalone lure, more applicable for use in urban areas where natural sources of host odour are less common.

### Conclusions

The results of this study demonstrate the feasibility of using a synthetic pheromone lure to attract *L. longipalpis* over a prolonged period of time, and therefore the viability of this approach for sand fly control. Further studies will be required to determine the extent to which this novel technology can reduce leishmaniasis transmission, and the general applicability of this approach for combating other insect-borne neglected diseases.
